# Modelling neurodegeneration and inflammation in early diabetic retinopathy using 3D human retinal organoids

**DOI:** 10.1007/s44164-024-00068-1

**Published:** 2024-03-25

**Authors:** Luisa de Lemos, Pedro Antas, Inês S. Ferreira, Inês Paz Santos, Beatriz Felgueiras, Catarina M. Gomes, Catarina Brito, Miguel C. Seabra, Sandra Tenreiro

**Affiliations:** 1 iNOVA4Health, NOVA Medical School|Faculdade de Ciências Médicas, NMS|FCM, Universidade Nova de Lisboa, Rua Camara Pestana, 6, Lisbon, Portugal; 2 UCL Institute of Ophthalmology, London, UK; 3 iBET, Instituto de Biologia Experimental E Tecnológica, Apartado 12, 2781-901 Oeiras, Portugal; 4 Instituto de Tecnologia Química E Biológica António Xavier, Universidade Nova de Lisboa, Avenida da República, 2780-157 Oeiras, Portugal

**Keywords:** Diabetic retinopathy, Retinal organoids, Retinal degenerative diseases, Human neuroretina, Hyperglycaemia

## Abstract

**Purpose:**

Diabetic retinopathy (DR) is a complication of diabetes and a primary cause of visual impairment amongst working-age individuals. DR is a degenerative condition in which hyperglycaemia results in morphological and functional changes in certain retinal cells. Existing treatments mainly address the advanced stages of the disease, which involve vascular defects or neovascularization. However, it is now known that retinal neurodegeneration and inflammation precede these vascular changes as early events of DR. Therefore, there is a pressing need to develop a reliable human in vitro model that mimics the early stage of DR to identify new therapeutic approaches to prevent and delay its progression.

**Methods:**

Here, we used human-induced pluripotent stem cells (hiPSCs) differentiated into three-dimensional (3D) retinal organoids, which resemble the complexity of the retinal tissue. Retinal organoids were subjected to high-glucose conditions to generate a model of early DR.

**Results:**

Our model showed well-established molecular and cellular features of early DR, such as (i) loss of retinal ganglion and amacrine cells; (ii) glial reactivity and inflammation, with increased expression of the vascular endothelial-derived growth factor (*VEGF*) and interleukin-1β (*IL-1β*), and monocyte chemoattractant protein-1 (MCP-1) secretion; and (iii) increased levels of reactive oxygen species accompanied by activation of key enzymes involved in antioxidative stress response.

**Conclusion:**

The data provided highlight the utility of retinal organoid technology in modelling early-stage DR. This offers new avenues for the development of targeted therapeutic interventions on neurodegeneration and inflammation in the initial phase of DR, potentially slowing the disease’s progression.

**Supplementary Information:**

The online version contains supplementary material available at 10.1007/s44164-024-00068-1.

## Introduction

Diabetic retinopathy (DR) is a common complication of diabetes and a leading cause of vision loss worldwide [1–3]. The traditional model of DR as a microvascular disease has evolved. It is now well-established that neurodegeneration is not only significant in DR but is also considered an early event, preceding visible microvascular abnormalities. This fact is referred to as diabetic retinal neurodegeneration (DRN) [4–6]. However, there are no treatments targeting the early stages of the disease before the onset of vascular defects such as macular edema or neovascularization or before retinal neurodegeneration occurs [7–9]. Although reactive gliosis, neural apoptosis, and microvascular dysfunction can be interdependent and essential for developing DR, neuroprotection can be considered a therapeutic target per se [4, 5]. Consequently, it is of paramount importance to elucidate the intricate molecular and cellular mechanisms underpinning DRN and to develop novel therapeutic approaches targeting its early stages, thereby mitigating the disease’s progression.

Hyperglycaemia, hypertension and diabetes duration are considered the established risk factors for developing DR [10–12]. Several cellular pathways have been proposed to explain DR. The most studied mechanisms are increased polyol and hexosamine pathways flux, increased advanced glycation end-products (AGE) formation, abnormal activation of protein kinase C (PKC) pathway, and increased oxidative stress [10]. Common grounds for all these mechanisms are oxidative stress, inflammation, vascular occlusion, upregulation of factors such as insulin-like growth factor (IGF), vascular endothelial growth factor (VEGF), tumour necrosis factor (TNF), and basic fibroblast growth factor-2 (bFGF). The activation of these pathways leads to the degeneration of the neural retina and capillary abnormalities in the inner retina. Neural apoptosis and reactive gliosis are considered the most important histological features of DR. Retinal ganglion cells (RGCs), located in the inner retina, are the more susceptible cells to hyperglycaemia [13], and RGCs loss has been detected in diabetic rats and diabetic patients either without or with only minimal DR [13–18]. In addition to RGCs, amacrine cells, and photoreceptors have shown an increased apoptotic rate in diabetic retinas [18–20].

In the last decades, in vitro two-dimensional (2D) models of DR have contributed to characterize the cellular processes of retinal damage during diabetic conditions. These models provide simpler systems of one or two cell types in interaction for studying the cytotoxic effects of high glucose, glutamate, or AGEs [21]. Particularly, cell cultures of dorsal root ganglion cells, cortical and hippocampal neurons, and RGCs were frequently used to highlight the early events in DR [22–24]. The involvement of Müller glia in DR was also explored on primary Müller cells and established Müller cell lines and have contributed to understanding the proinflammatory role of Müller cells in high-glucose conditions [25–27].

Organotypic retinal cultures, or retinal explants, which preserve the histotypic architecture of the retina have been established to elucidate the interplay between neuronal and glial compartments in the context of DR. Nevertheless, these models pose challenges in regard to duration and the viability of the in vitro culture [21, 28, 29].

Up to now, several animal models have been proposed to study the molecular and cellular mechanisms of DR, such as inducted hyperglycaemia, spontaneously diabetic rodents, genetic DR models carrying mutations in the leptin or insulin-2 genes, alongside models of angiogenesis without diabetes [30–33]. However, rodent models inherently possess distinct disparities when compared with human retinas. Key differences include the lack of a cone-rich macula, different distribution, and types of photoreceptor cells, as well as different proportions of other retinal cellular subtypes [34]. Due to these differences, the direct translation of findings derived from animal models to humans is limited [35].

The advent of stem cell-based models and cell technologies has opened new avenues for basic research and clinical applications. In the retina field, the development of three-dimensional (3D) retinal organoids derived from pluripotent embryonic stem cells is at the forefront of these efforts [36–38]. Specifically, retinal organoids derived from human induced pluripotent stem cells (hiPSCs) largely resemble the human tissue architecture and recapitulate the cellular interactions, which is crucial to the development and function of their in vivo counterparts [35, 39–42]. Organoids are emerging not only to be an effective model in the translational arena but also a valuable tool for understanding human development, physiology, and disease [43, 44]. In recent years, retinal organoids have been used as models for several purposes such as to identify the mechanisms of inherited retinal degenerative diseases [45], to test advanced therapies based on adeno-associated virus (AAV) infection for retinal cells [46, 47], for RNA-based therapies [48], to study retinal viral infections such as severe acute respiratory syndrome coronavirus 2 (SARS-CoV-2) [49], and as a source of human cone photoreceptors for cell replacement therapies [50]. Moreover, retinal organoids are also being explored in novel microphysiological systems (MPS) involving retina-on-chip co-culture approaches [51].

To the best of our knowledge, we have established the first 3D human retinal organoid model to study the early molecular mechanisms in DR. We cultured retinal organoids derived from hiPSCs under high-glucose conditions to mimic the retinal environment in DR. We observed retinal neurodegeneration, specifically in retinal ganglion and amacrine cells, and an inflammatory response accompanied by the expression of interleukin-1β (*IL-1β*), *VEGF* and secretion of monocyte chemoattractant protein-1 (MCP-1). Additionally, we found increased reactive oxygen species (ROS) levels and activation of the antioxidative stress response and mTOR pathway. Our findings show that our 3D human retinal model successfully replicates several disease features of DRN, serving as an innovative platform for investigating DR mechanisms and pinpointing potential therapeutic targets. This approach offers a compelling preclinical model of early-stage DR, aligning with the 3Rs principle and the call for alternative and non-animal research methodologies. Our methodology is poised to reduce reliance on animal models, enabling data prioritisation for subsequent validation and diminishing risks associated with data translation to human applications.

## Materials and methods

### Human-induced pluripotent stem cell (hiPSC) culture

hiPSC line (IMR90-4, WiCell) derived from a healthy donor and fully characterized was cultured with mTeSR™ Plus Medium (STEMCELL Technologies) on growth factor–reduced matrigel-coated 6-well plates (Corning) and used for all experiments in this study. The hiPSCs were routinely passaged using Versene (Gibco, Thermo Fisher Scientific) at a ratio of 1:4–1:6 in mTeSR™ Plus medium supplemented with 10 µM of ROCK inhibitor Y-27632 (Focus Biomolecules) and maintained at 37 °C in a humidified atmosphere containing 5% CO_2_.

### Retinal organoid differentiation of hiPSCs

The method for hiPSCs differentiation towards retinal organoids was carried out according to a previously described protocol [42] with slight modifications to improve the efficiency and reproducibility in the generation of retinal organoids. Briefly, hiPSCs at 80% of confluence were lifted using Versene (Gibco, Thermo Fisher Scientific) and split at 3000 cells/well in a Nunclon Sphera 96-well U-bottom plate (Thermo Fisher Scientific) using mTeSR™ Plus medium supplemented with 10 µM of ROCK inhibitor (Focus Biomolecules). The cells were grown as aggregates/embryoid bodies for 6 days, doing an adaptation to Neural Induction Medium (NIM) containing DMEM/F12, 1% N2 supplement, 1 × non-essential amino acids, 1 × GlutaMax (all from Gibco, Thermo Fisher Scientific), and 2 mg/ml Heparin (Sigma-Aldrich). On day 6, 1.5 nM of bone morphogenic protein-4 (BMP4, Peprotech) was added to fresh NIM, and on day 7, EBs were transferred to growth factor–reduced matrigel-coated 6-well plates (Corning). The medium was replaced by half-fresh NIM on days 9, 12, and 15. On day 16, the medium was replaced by retinal differentiation medium (RDM) containing DMEM:F12 (3:1), 2% B27 minus vitamin A, 1 × non-essential amino acids, 1 × GlutaMax, 1 × antibiotic/antimycotic (all form Gibco, Thermo Fisher Scientific), and changed every 2–3 days. At day 30, optic vesicles were manually dissected using a surgical scalpel (SM65A, Swann-Morton Ltd) under the microscope EVOS XL core (Thermo Fisher Scientific). After dissection, organoids were maintained in suspension flasks (Sarstedt) in 3D-retinal differentiation medium (3D-RDM) containing DMEM:F12 (3:1), 2% B27 minus vitamin A, 1 × non-essential amino acids, 1 × GlutaMax, 1 × antibiotic/antimycotic, 5% FBS, 1:1000 chemically defined lipid supplement (all from Gibco, Thermo Fisher Scientific), 100 µM taurine (Sigma-Aldrich), and 1 µM of all-trans retinoic acid (RA, Sigma-Aldrich) until day 100. After this differentiation stage, organoids were maintained in 3D-RDM and processed for high-glucose treatments.

### High-glucose treatments

Organoids on day 100 were exposed to different glucose concentrations. Namely, the 3D-RDM medium (containing a standard glucose concentration of 19 mM) was supplemented with D-glucose (Sigma-Aldrich) to a final concentration of 50 mM and 75 mM. D-mannitol (Sigma-Aldrich) was used as osmotic control (56 mM of mannitol was added to the 19 mM of glucose already present in the 3D-RDM medium to obtain an equivalent osmolality as in the highest glucose condition, 75 mM). Control organoids were maintained in a regular 3D-RDM medium with 19 mM of glucose. Organoids were maintained for 6 days, with fresh medium exchange every other day. After treatment, organoids were then collected for further analysis.

### Measure of intracellular ROS levels

ROS was measured using the cell-permeable fluorogenic probe 2′,7′-Dichlorodihydroflurescein diacetate (DCF-DA, Sigma-Aldrich), widely used to determine the degree of overall oxidative stress. Briefly, after glucose treatment, organoids were incubated in a 3D-RDM medium with 20 µM of DFC-DA for 1 h at 37 °C. Positive control was done using 0.5% H_2_O_2_ for 30 min. After incubation, organoids were rinsed once in PBS and plated in chamber slides for live imaging using a confocal microscope Zeiss LSM 980 (Zeiss). The mean fluorescence intensity per image was determined using Fiji (Image J) software.

### Glutamine release assay

The glial function was quantified as an intracellular conversion of L-glutamate to L-glutamine [52]. L-glutamic acid (Sigma) was added at 3 mM in GlutaMax-free 3D-RDM medium on the third day of glucose treatment. Medium culture samples were collected on day 6, centrifugated at 200* g*, 4 °C for 10 min, and supernatants were collected for glutamine analysis. GlutaMax-free 3D-RDM medium without L-glutamic acid was used as a negative control for determining the assay background. Glutamine quantifications were measured using Cedex Bio analyser 7100 (Roche), and concentrations were normalized by the protein content of each sample (mmol/L/µg).

### Histology and immunofluorescence

Retinal organoids were fixed in 4% paraformaldehyde phosphate-buffer solution (PFA, Sigma) for 20 min at room temperature. Washed twice with phosphate-buffered saline (PBS) and incubated in 10% for 1 h, 20% for 1 h, and 30% sucrose solution overnight at 4 °C. Retinal organoids were placed into cryogenic moulds and immersed in optimum cutting temperature (OCT Cryomatrix, Fisher Scientific). About 12 µm slices were sectioned on a Leica CM3050 S cryostat (Leica). Cut sections were permeabilized with 0.05% Triton-X-100 (Sigma-Aldrich) in PBS for 15 min and were blocked in 10% Donkey Serum (DS) in PBS for 1 h. Primary antibodies were incubated in a blocking solution at 4 °C overnight. The following primary antibodies were used: mouse anti-AP2α (1:100, sc-12726, Santa Cruz Biotechnology), goat anti-BRN3a (1:100, sc-31989, Santa Cruz Biotechnology), mouse anti-CRX (1:200, H00001406-M02, Abnova), rabbit anti-gamma Synuclein/*SNCG* (1:200, ab52633, Abcam), goat anti-OTX2 (1:100, AF1979, R&D Systems), rabbit anti-vimentin (1:200, 5741, Cell Signalling), and Alexa Fluor 568 Phalloidin (1:400, A12380, Invitrogen). Sections were then washed three times with PBS followed by incubation of secondary antibodies for 1 h at room temperature. The following secondary antibodies were used (all from Invitrogen and used at 1:1000 dilution): donkey anti-goat 488 (A11055), donkey anti-mouse 488 (A21202), and donkey anti-rabbit 488 (A21206). Sections were then incubated with 4′,6-diamidino-2-phenylindole (DAPI, 1 µg/ml, Sigma-Aldrich), washed twice with PBS, and mounted with Vectashield-mounting medium (Vector Laboratories). Confocal images were acquired using Zeiss LSM 980 (Zeiss), and images were processed using ImageJ Software. Automatic fluorescence intensity quantification of vimentin and cell counting analysis was performed using the specific ImageJ plugins (at least five sections per condition were analysed from three independent experiments). For the quantitative cell-counting analysis, the number of retinal populations’ (specifically RGCs, amacrine cells, and photoreceptor progenitors) positive cells were expressed relative to the total number of DAPI-stained nuclei.

### Fluoro-Jade C staining

Fluoro-Jade C is an anionic fluorochrome commonly used for identifying degenerating neurons in the brain and in the retina regardless of the neurotoxic insult or the mechanism of cell death [53–55]. Fluoro-Jade C labelling was performed on retinal organoids cryosections using the Fluoro-Jade® C staining kit (Biosensis) following the manufacturer’s instructions. After co-incubation of Fluoro-Jade C and 4′,6-diamidino-2-phenylindole (DAPI), slides were air-dried for 10 min, cleared in xylene, and mounted with Entellan™ (Sigma-Aldrich) mounting media. Samples were analysed in the confocal microscope Zeiss LSM980 (Zeiss), and Fluoro-Jade C^+^ cells of each section were evaluated. Quantitative analysis was performed using ImageJ Software, and Fluoro-Jade C^+^ cell counting was expressed relative to the total number of DAPI-stained nuclei (five sections per condition were analysed from three independent experiments).

### Western blot analysis

Pools of 3 organoids were resuspended in cold lysis buffer (Cell Signaling Technology) supplemented with protease and phosphatase inhibitor cocktails (Roche) and sonicated twice for 3 s at 10% intensity in the Branson Digital Sonifier SFX 150 (Emerson). Lysates were then centrifuged at 7500 g for 10 min at 4 °C, and supernatants were collected for protein quantification using the Pierce BCA protein assay kit (Thermo Scientific) following the manufacturer’s instructions. A total of 20 µg of protein from each lysate was resolved on 10% or 12% sodium dodecyl sulphate—polyacrylamide gels (SDS-PAGE) and subsequently transferred to nitrocellulose membranes (Bio-Rad Laboratories). Membranes were blocked using 5% non-fat dry milk or 5% bovine serum albumin (BSA) (Sigma-Aldrich) in Tris-buffered saline (TBS) (50 mM Tris, 150 mM NaCl, pH = 7.6) containing 0.1% Tween-20 (Sigma-Aldrich) (TBS-T). Primary Antibodies were incubated in a blocking solution overnight at 4 °C. The following primary antibodies were used: rabbit anti-glutamine synthetase (1:1000, NB110-41404, Novus Biologicals), mouse anti-catalase (1:500, sc-271803, Santa Cruz Biotechnology), mouse anti-SOD1 (1:1000, sc-31989, Santa Cruz Biotechnology), rabbit anti-SOD2 (1:1000, ab13533, Abcam), rabbit anti-phospho-Akt Ser473 (1:1000, 9271, Cell Signaling Technology), rabbit anti-Akt (1:1000, 9272, Cell Signaling Technology), rabbit anti-phospho-S6 ribosomal protein Ser235/236 (1:1000, 4858, Cell Signaling Technology), rabbit anti-S6 Ribosomal Protein (1:1000, 2217, Cell Signaling Technology), and mouse anti-β-Actin-peroxidase (1:25000). After washing with TBS-T, the appropriate HRP-conjugated secondary antibody was added (1:5000 in blocking buffer) for 2 h at room temperature. Secondary antibodies used are the donkey anti-rabbit HRP (NA934, Cytiva) and sheep anti-mouse HRP (NA931, Cytiva). Antibody binding was detected using chemiluminescence ECL Prime Western Blotting Substrate (Cytiva), and images were acquired on ChemiDoc Touch (Bio-Rad Laboratories). The acquired images were processed and quantified using Image Lab software (Bio-Rad laboratories), and the protein of interest was normalized using β-Actin as a loading control.

### RNA extraction and quantitative real-time PCR

Total RNA from a pool of 3 organoids was isolated using the RNeasy mini kit (Qiagen) according to the manufacturer’s protocol. Overall, 1 µg of mRNA was reverse transcribed into cDNA using a Superscript II reverse transcriptase kit (Invitrogen). The cDNA samples were used for quantitative PCR in the LightCycler 96 system (Roche) using the FastStar Essential DNA Green Master (Roche) following the manufacturer’s instructions. Primer pairs were designed using Primer-BLAST (NCBI) and synthesized by Thermo Fisher Scientific. The following primer sequences were used (forward/reverse): *AKT*, (5′-TGATCACCATCACACCACCT-3′/5′-CTGGCCGAGTAGGAGAACTG-3′); *GPX*, (*5′-*TGGGCATCAGGAGAACGCCA-3′/5′-GCGTAGGGGCACACCGTCAG-3′); *HIF1⍺*, (5′-CAGTCGACACAGCCTGGATA-3′/5′-GCGGCCTAAAAGTTCTTCTG-3′); *IL1B*, (5′-GTTTCTCTGCAGAAAGAGGC-3′/5′-AATGCCAGAGATGCATTGG-3′); *mTOR*, (5′-CTGGTTTCACCAAACCGTCT-3′/5′-GCACGACGTCTTCCAGTACC-3′); *SOD1*, (5′-TGGCCGATGTGTCTATTGAA-3′/5′-ACCTTTGCCCAAGTCATCTG-3′);* SOD2*, (5′-TGGTTTCAATAAGGAACGGG-3′/5′-GAATAAGGCCTGTTGTTCCT-3′); *VEGF*, (5′-CCTTGCTGCTCTACCTCCAC-3′/5′-ATGATTCTGCCCTCCTCCTT-3′); *β-Actin*, (5′-GAAGATCAAGATCATTGCTCCTC-3′/5′-ATCCACATCTGCTGGAAGG-3′). The expression levels were normalized to the housekeeping β-Actin and fold change was calculated using the 2^−∆∆Ct^ method.

### Enzyme-linked immunosorbent assay (ELISA)

MCP-1 levels secreted by organoids were assessed using the Human MCP-1 Standard TMB ELISA development kit (Peprotech) according to the manufacturer’s protocol. Briefly, cell supernatants were plated in duplicates and incubated with MCP-1 detection antibody and HRP-streptavidin conjugate. After stopping colour development reaction, the absorbance was measured at 450 nm using a Synergy HT microplate reader (Agilent) and normalized by the protein content of each sample ((ng/ml)/ µg of protein).

### Data analysis and statistics

Statistical analyses between treatment groups and controls were performed using Prism 8 (GraphPad Software). Data are presented as the mean ± SD, and statistical tests were conducted using the one-way ANOVA followed up by Tukey’s multiple comparison test. All statistical comparisons were performed on data from ≥ 3 biologically independent experiments. Significance is shown as *p < 0.05; **p < 0.01; ***p < 0.001.

## Results

### High-glucose treatment induces neurodegeneration in retinal organoids

**Fig. 1:**
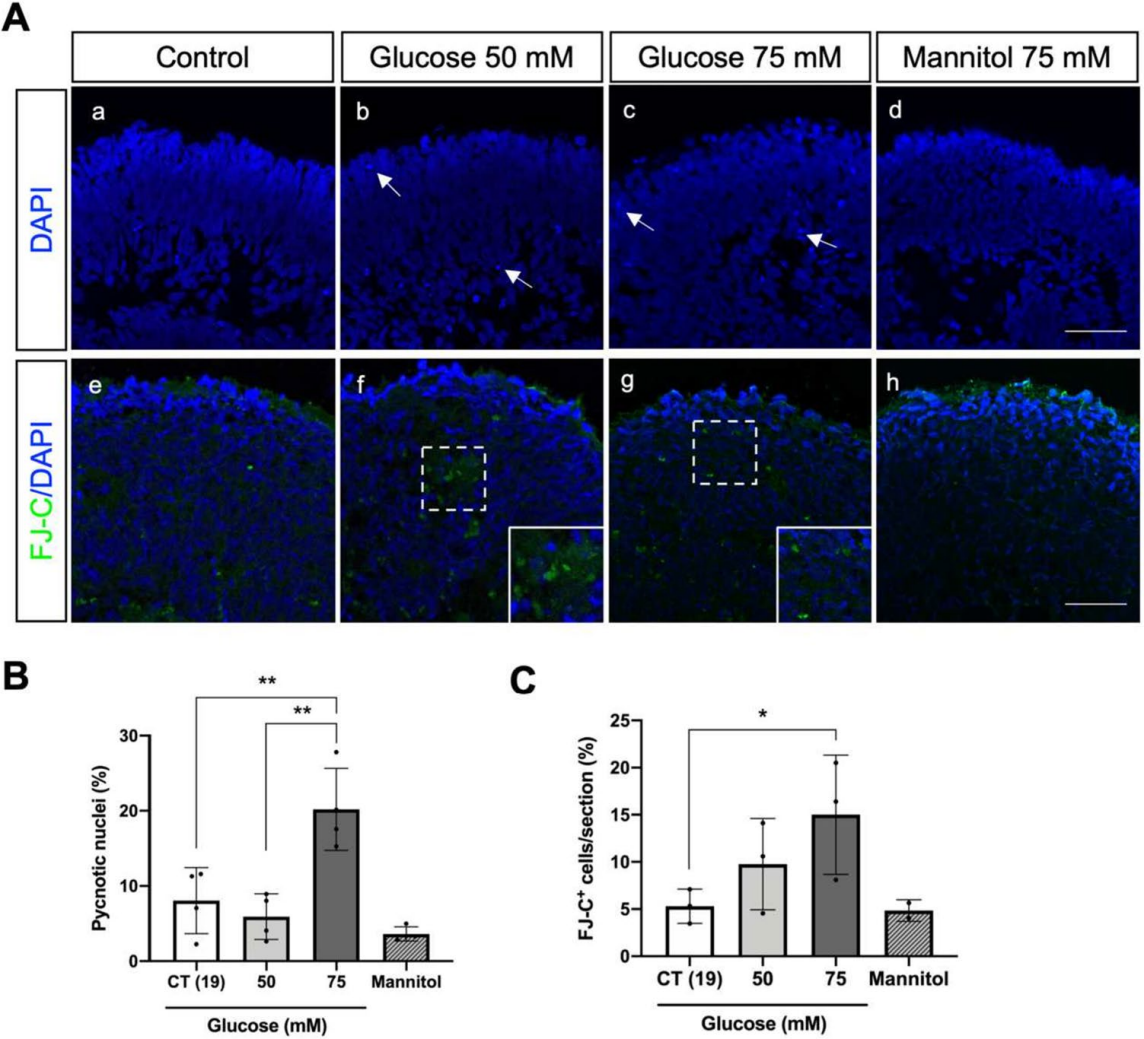
High-glucose treatment induces neurodegeneration of retinal organoids. **A** Cross-sectional images of retinal organoids with DAPI nuclei staining (in blue) showing pyknotic nuclei (a–d) and (e–h) represent cross-sectional images of retinal organoids with FluoroJade-C (FJ-C) staining (in green). White arrows indicate highly condensed pyknotic nuclei and white boxes indicate a zoom of degenerating neurons. Scale bar—50 µm. **B** Quantification of pyknotic nuclei after high glucose treatment. **C** Quantification of FJ-C positive cells (FJ-C.^+^) after high glucose treatment. Statistical results represent the mean values ± SD from at least 3 independent differentiations, in which 3 organoids from each condition were used for analysis (*p-value < 0.05; **p-value < 0.01)

For this study, retinal organoids were generated from hiPSC line IMR90-4 according to the three-stage differentiation protocol described by Capowski et al. [42]. We used retinal organoids differentiated for 100 days. At this stage, these organoids contain pivotal retinal cells affected in DR (Supplementary Fig. 1). Specifically, (i) most cells of the neuroretina are present, such as RGCs located in the basal region; (ii) photoreceptor progenitors are found in the apical layers, and starburst amacrine cells are also identified [42, 56]; (iii) Müller glia progenitors span the entire width of the organoid, mirroring the distribution of Müller cells in the human retina [57]; and (iv) the 100-day organoids display a complete lamination of the inner retina [42]. Given these features, retinal organoids at 100 days of differentiation offer a physiologically relevant model for disease modelling (Mahato et al., 2022), drug testing, and even cell transplantation [57]. First, we exposed retinal organoids with 100 days of differentiation to either 19 mM of glucose (standard glucose concentration in the 3D-RDM medium) or high-glucose concentrations (50 and 75 mM) for 6 days. The nuclei staining with DAPI of cross-sections revealed a significant increase in the percentage of pyknotic nuclei on the organoids exposed to 75 mM of glucose in comparison with control and 50 mM of glucose (Fig. 1A (images a–c) and B). Furthermore, mannitol-treated organoids did not show significant differences in the number of pyknotic nuclei in comparison with the control condition (Fig. 1A (image d) and B). In parallel, we evaluated neurodegeneration induced by high-glucose conditions by assessing the percentage of positive cells for Fluoro-Jade-C (FJ-C) staining, which is considered a reliable marker of degenerating immature and mature neurons, including apoptotic, necrotic, and autophagic cells [58]. We observed a significantly increased percentage of FJ-C positive cells in cross-sections of organoids exposed to 75 mM compared to the control condition (Fig. 1A (images e–h) and C).

Overall, these data show that the exposure of the retinal organoids to high glucose induces cell death and neurodegeneration, as reported in the early stages of DR in both animal models and in the eyes of diabetic patients [4–9, 18].

### High-glucose treatment induces retinal ganglion and amacrine cell loss in retinal organoids

**Fig. 2:**
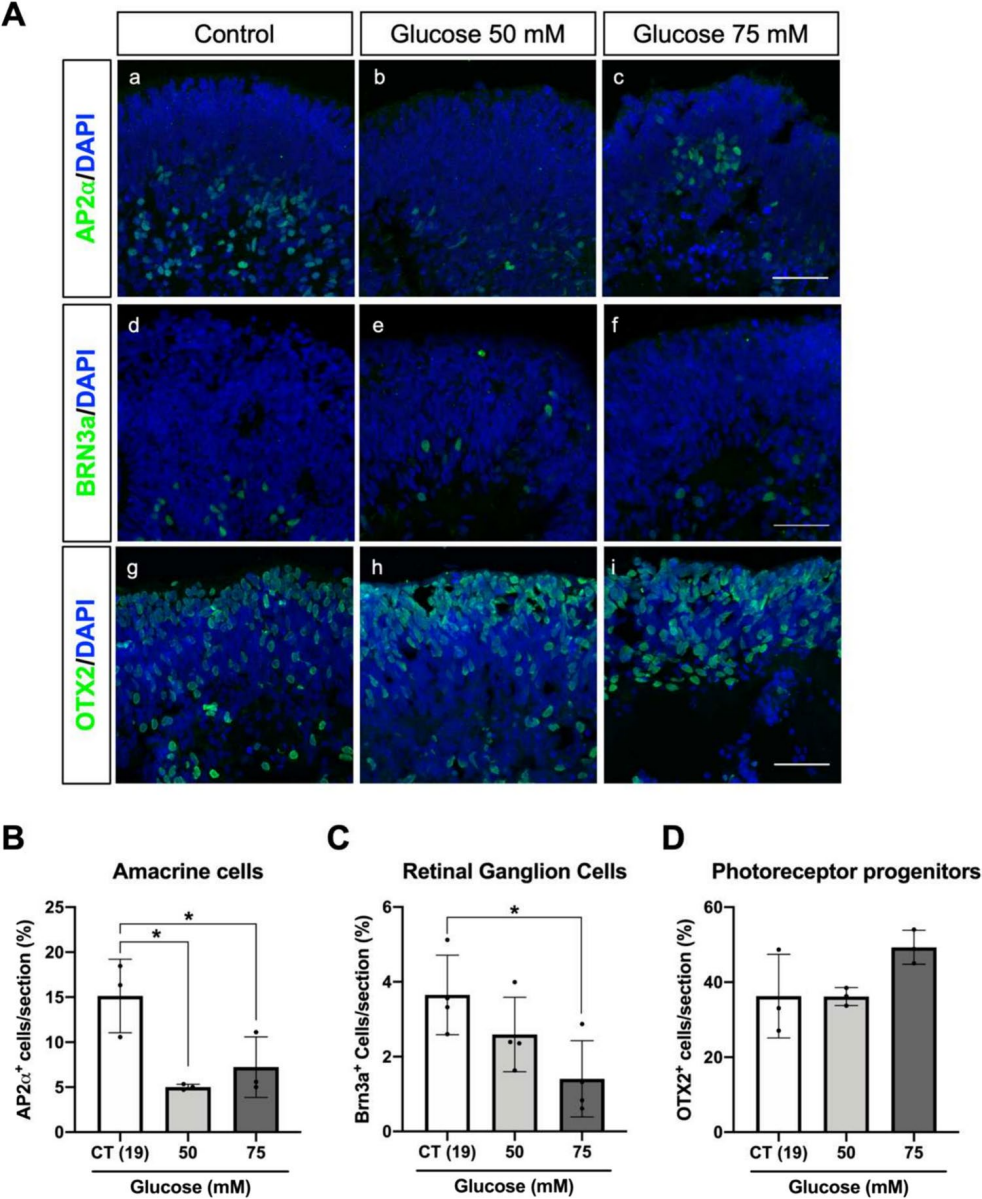
High-glucose treatment induces amacrine and retinal ganglion cell loss. **A** Immunofluorescence analysis of organoids subjected to high-glucose (a–c) staining of AP2⍺ (amacrine cells), (d–f) staining of BRN3a (retinal ganglion cells) and (g–i) staining of OTX2 (photoreceptor progenitors). Organoid sections were counterstained with DAPI. Scale bar—50 µm. **B** Quantification of AP2⍺-positive cells counted per field of view. **C** Quantification of Brn3a-positive cells counted per field of view. **D** Quantification of OTX2-positive cells counted per field of view. Data represent mean ± SD of at least 3 independent differentiations, in which 3 organoids from each condition were used for analysis (*p-value < 0.05)

Several studies performed in human retinal sections from diabetic patients have shown that RGCs are amongst the most affected retinal cells in DRN. Amacrine and photoreceptor cell death has also been observed in retinal sections [13, 18–20]. We evaluated how high-glucose exposure affected the different retinal cell types of the retinal organoids. AP2α belongs to a family of AP2 transcription factors and is commonly used as a marker of amacrine cells. However, AP2α is also known to be expressed in developing horizontal cells [59, 60], as is the case of retinal organoids at day 100 of differentiation. Indeed, the number of AP2α positive cells per section decreased by 50% in retinal organoids exposed to high-glucose conditions, indicating a reduction of amacrine and horizontal cells (Fig. 2A (images a–c) and B). Moreover, the number of BRN3a positive cells (RGCs) per section presented a significant twofold decrease at higher glucose conditions (75 mM) (Fig. 2A (images d–f) and C), while the number of OTX2 positive cells (photoreceptor progenitor cells) has not changed (Fig. 2A, images g-i and 2D). This data reinforces that treating retinal organoids for 6 days with high-glucose concentrations is enough to reproduce the loss of specific populations observed in DRN.

### High-glucose treatment induces inflammation in retinal organoids

**Fig. 3:**
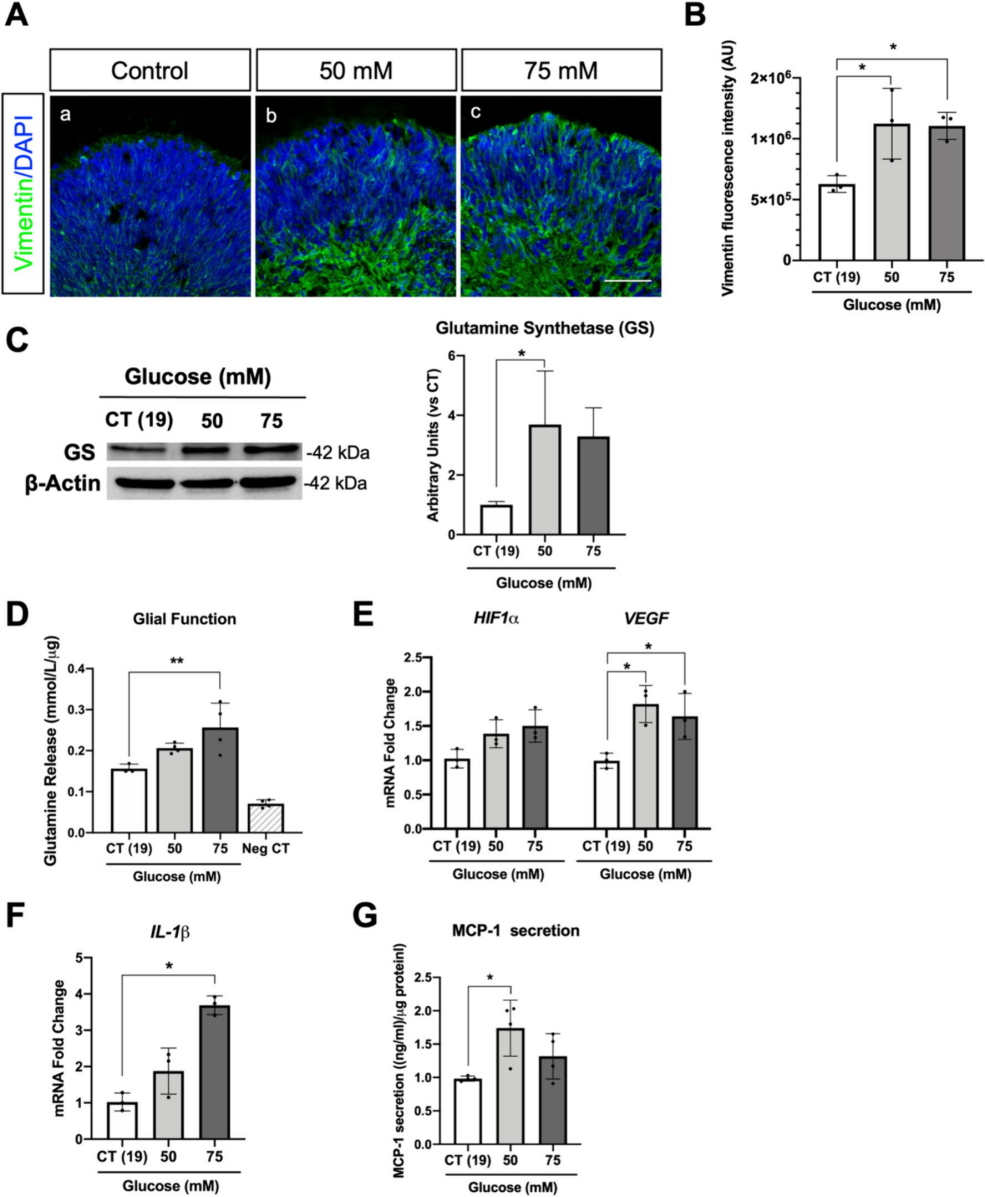
High-glucose treatment induces an inflammatory response in retinal organoids. **A** Immunofluorescence staining of vimentin (a–c) counterstained with DAPI (blue). Scale bar—50 µm. **B** Confocal image quantification of vimentin fluorescence intensity. **C** Western blot analysis of glutamine synthase (GS) (left panel) and correspondent densitometry analysis of GS normalized by β-Actin. **D** Glutamine release in the culture medium after high-glucose treatment, reflecting the conversion of glutamate to glutamine by the glial cells. GlutaMax-free 3D-RDM medium was used as negative control **E** Expression of *HIF1⍺* and *VEGF* mRNA. **F** Expression of the pro-inflammatory cytokine *IL-1β* mRNA. **G** MCP-1 secretion levels in the culture medium measured by ELISA. Results represent the mean ± SD of at least 3 independent differentiations, in which 3 organoids from each condition were used for analysis (*p-value < 0.05; **p-value < 0.01)

Müller cells, the principal glial cells in the retina, are thought to be a major source of inflammatory factors in DR [61, 62]. Müller cells express and secrete several growth factors and cytokines that alter the function and survival of retinal neurons and capillary cells. These cells could be responsible for the production and secretion of several inflammatory mediators including VEGF, MCP-1, tumour necrosis factor-α (TNF-α), IL-1β, and interleukin-6 (IL-6) [61]. Functional Müller cells contribute to the removal of the extracellular glutamate and the production of glutamine essential for retinal neurons. Under pathological conditions, such as glaucoma or ischemia, the dysregulation of Müller cells showed a decrease in the glutamate uptake and the glutamine release caused by the impairment of glutamine synthetase [63–65]. In contrast, in DR and optic nerve crush, no changes or a slight increase of glutamine synthetase is observed [62, 66]. Our results showed, that at day 100, retinal organoids present Müller glia progenitors as indicated by immunostaining of cross-sections with the corresponding marker vimentin (Fig. 3A). Interestingly, vimentin immunostaining signal increases significantly and cytoskeleton morphology changes are observed under high-glucose treatment (Fig. 3A and B). Additionally, the protein levels of glutamine synthetase were increased fourfold in organoids exposed to 50 mM compared to the control conditions (Fig. 3C) suggesting an increase in glial function. Moreover, the levels of glutamine release were significantly increased in organoids exposed to 75 mM of glucose compared to the control conditions (from approximately 0.15 to 0.25 mmol/L/µg) (Fig. 3D). The increase of glutamine synthetase may be attributed to an increase of the defence against oxidative stress and as a proactive response to safeguard against neuronal degeneration under high-glucose conditions [67]. Furthermore, considering the pivotal role of hypoxia-inducible factor 1*α* (*HIF-1α*) in regulating cellular oxygen homeostasis and aiding adaptation to hypoxia during DR pathogenesis—along with its role in the expression of pro-inflammatory cytokines and VEGF [68]—we decided to assess the expression of *HIF-1α* and *VEGF*. Our results showed that *HIF-1α* expression does not change. However, *VEGF* expression was almost at twofold increase in retinal organoids under high-glucose treatment (Fig. 3E). In addition, the pro-inflammatory cytokine *IL-1β* expression was increased when treated with 75 mM of glucose. (Fig. 3F). Moreover, MCP-1 secretion is known to be produced by glial cells in diabetic patients [69]. Interestingly, we observed increased secretion of MCP-1 at 50 mM of glucose (Fig. 3G). Altogether, our results show that under our experimental design, retinal organoids at day 100 already present markers of gliosis and inflammation, which are essential features of DRN [61, 70].

### High-glucose treatment induces oxidative stress in retinal organoids

**Fig. 4:**
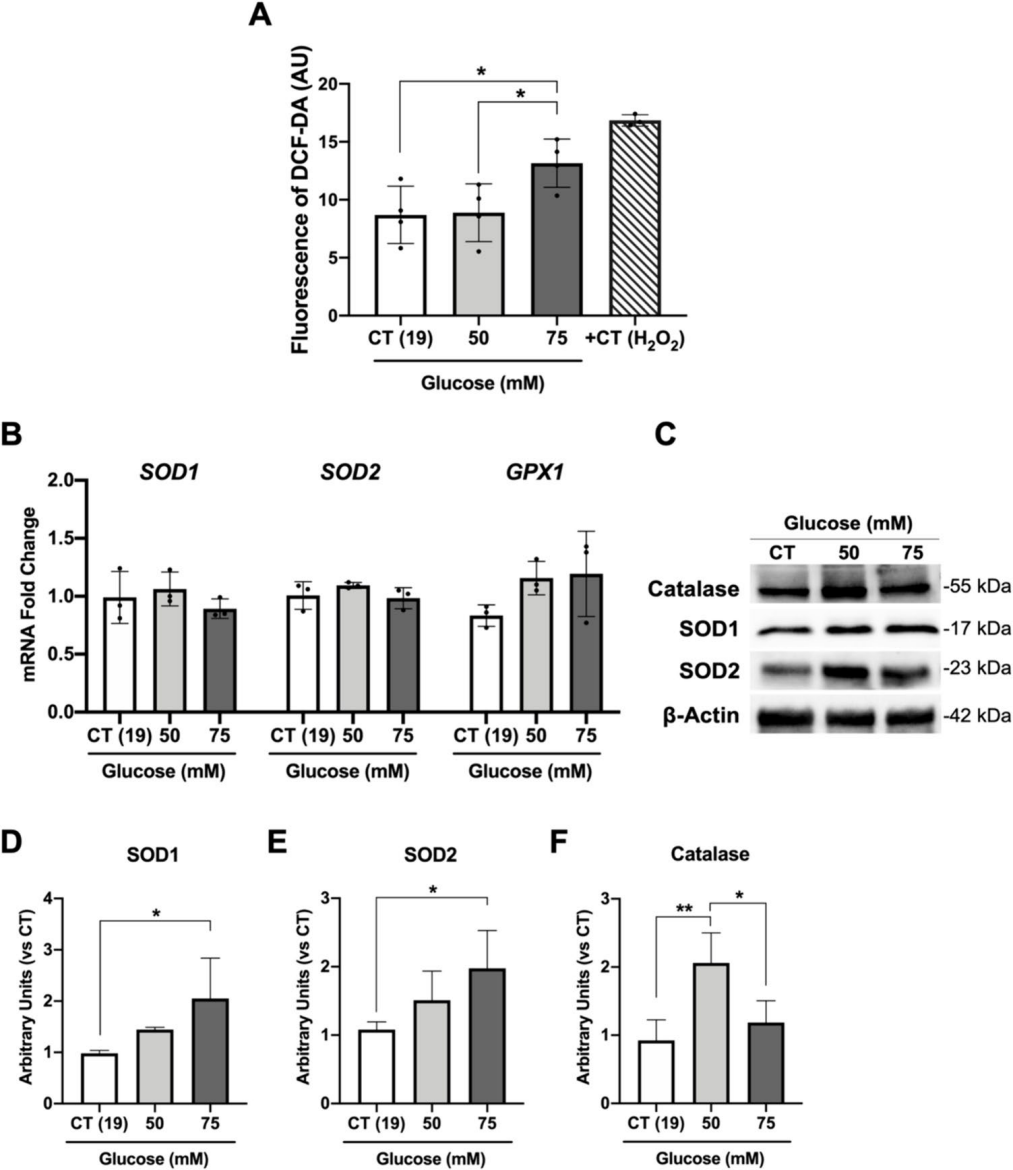
High-glucose treatment affects the antioxidant response in organoids. **A** Fluorescence intensity of 2,7-dichlorofluorescein diacetate (DCF-DA) probe for detection of reactive oxygen species in organoids after high glucose exposure (*n* = 4). **B** Expression of *SOD1*, *SOD2* and *GPX1* mRNA levels. **C** Western blot analysis of catalase and superoxide dismutases 1 and 2 (SOD1 and SOD2). **D–F** Densitometry analysis of SOD1, SOD2 and catalase normalized by β-Actin**.** Data represent the mean ± SD of at least 3 independent differentiations, in which 3 organoids from each condition were used for quantification (*p-value < 0.05; **p-value < 0.01)

Oxidative stress stands as a defining feature of DR. Under the chronic influence of elevated glucose levels, there is an increased production of ROS spurred by the activation of secondary pathways such as the polyol and hexosamine pathways. Furthermore, the overproduction of AGEs concurrently increases ROS production, thereby establishing a self-perpetuating cycle [71, 72]. To determine whether oxidative stress was induced in the experimental conditions used in this study, we first evaluated the production of ROS levels assessed by the 2′-7′-dichlorofluorescin diacetate (DCF-DA) probe. Using the fluorescence intensity of DCF-DA as captured by confocal imaging, we observed a notable increase in intracellular ROS production in organoids exposed to high-glucose conditions (75 mM) when compared to both the control and the 50 mM conditions. (Fig. 4A). Additionally, we examined the gene expression and protein levels of pivotal enzymes central to the primary defence against oxidative stress, including superoxide dismutases (SODs), catalase, and glutathione peroxidase 1 (GPX1). Notably, while our results revealed no changes in *SODs* expression levels (Fig. 4B), there was a significant increase in the protein levels of SOD1 and SOD2 in retinal organoids under high-glucose conditions. This suggests that post-transcriptional regulation of these enzymes is taking place as a consequence of ROS production (Fig. 4C–E). Moreover, no changes were observed in the expression of *GPX1* under high-glucose conditions (Fig. 4B). However, there was a notable increase in catalase protein levels at 50 mM conditions (Fig. 4C and F). Collectively, these findings suggest that an antioxidant response is activated in retinal organoids exposed to high-glucose concentrations, likely to counteract the effects of ROS.

### High-glucose treatment induces mTOR pathway in retinal organoids

**Fig. 5:**
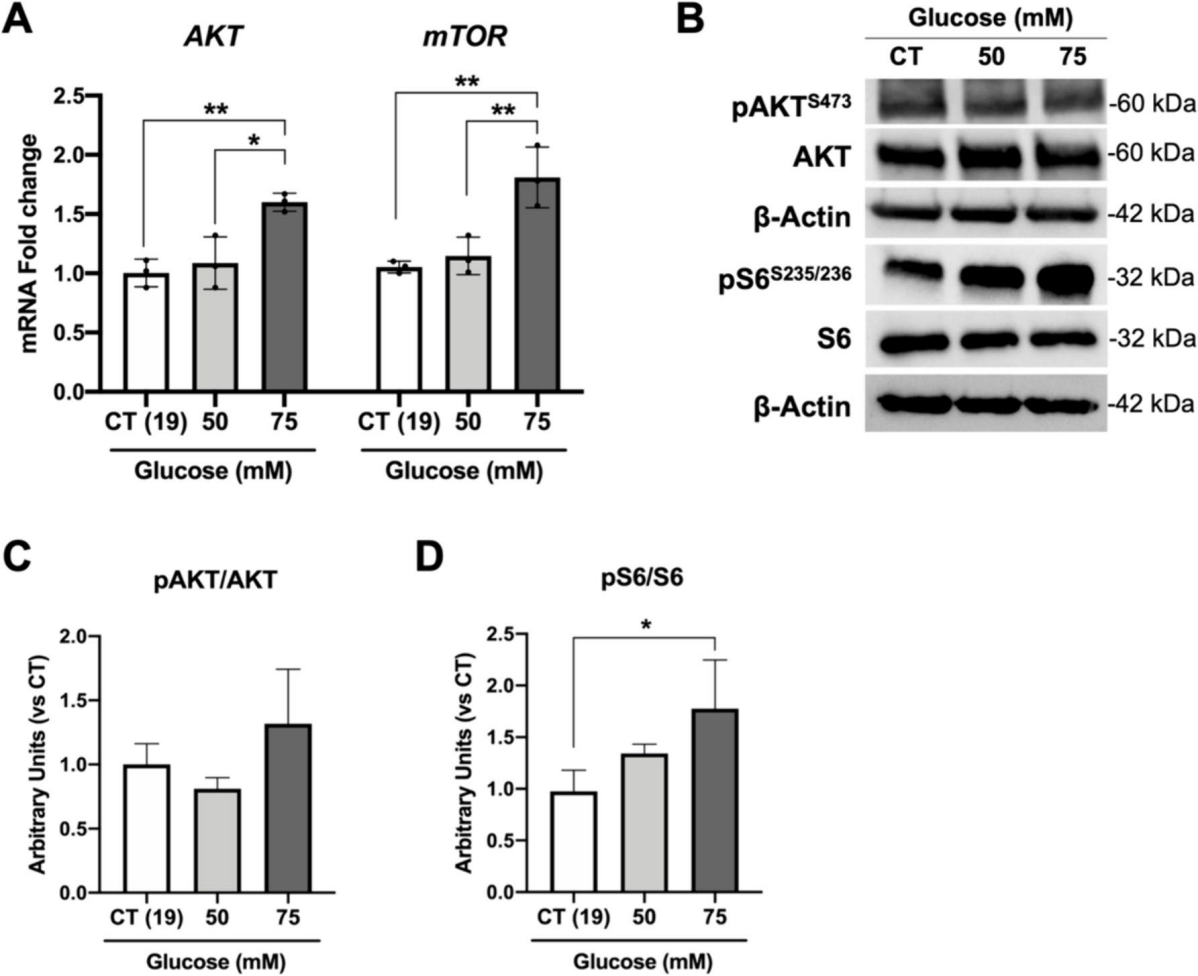
High-glucose treatment effects on mTOR signalling pathway. **A** Expression levels of *AKT* and *mTOR* mRNA levels after high-glucose treatment. **B** Western blot analysis of phospho-AKT^S473^, AKT, phospho-S6.^S235/236^ and S6 protein levels normalized by β-Actin. **C** Densitometry of pAKT/AKT levels after glucose treatment. **D** Densitometry of pS6/S6 protein levels after high-glucose conditions. Data represent mean ± SD of at least 3 independent differentiations, in which 3 organoids from each condition were used for quantification (*p-value < 0.05 and **p-value < 0.01)

The phosphatidylinositol 3-kinase (PI3K)/AKT/mammalian target rapamycin (mTOR) signalling pathway is a well-known central player in a large number of biological events related to cell growth, division, and metabolism [73]. Moreover, dysregulation in mTOR is associated with various diseases such as obesity, diabetes, cancer, and neurological diseases [74]. In DR, there is significant evidence that the PI3K/AKT/mTOR signalling pathway is connected to various mechanisms associated with disease progression, including oxidative stress, inflammation, hypoxia, angiogenesis, and proliferation [75, 76]. Therefore, we evaluated the expression levels of *mTOR* and *AKT.* A marked elevation in the expression of both genes was observed in retinal organoids subjected to 75 mM glucose, in contrast to the control (19 mM) and 50 mM treatment (Fig. 5A). Neither the AKT protein nor its S473 phosphorylation levels displayed significant changes under the conditions tested (Fig. 5B and C). Nevertheless, the phosphorylation levels of the mTOR’s primary downstream effector, the ribosomal S6 kinase (S6) at Ser235/236, showcased a significant increase in retinal organoids at 75 mM glucose when compared to the control and 50 mM treatment (Fig. 5B and D).

Together, these findings highlighted the activation of the mTOR signalling pathway in retinal organoids exposed to high levels of glucose.

## Discussion

The study and comprehension of any human disease are intimately dependent on the available experimental models. Both in vitro and in vivo DR models have been instrumental in clarifying the molecular and cellular mechanisms involved and also in identifying new therapies. Nevertheless, every existing DR model possesses its own set of advantages and drawbacks. Retinal organoids are emerging as powerful models to study retina development, diseases, toxicology, and new therapies [44, 45]. They can be derived from human adults or embryonic stem cells (ESCs) or hiPSCs obtained from healthy individuals or patients exhibiting specific diseases. In advanced stages of differentiation, these 3D structures effectively mimic all neuroretina cell types, including Müller cells and astrocytes, presenting them in an organized stratified layout reminiscent of the human retina. Moreover, these organoids can develop mature photoreceptors that are not only photosensitive but also capable of phototransduction, allowing advanced electrophysiological research [77, 78]. A shared trait amongst various organoid types, retinal ones included, is the absence of vascularization and microglia. Additionally, due to limited nutrient availability, there is an observed attrition of cells in the innermost retinal layer of the organoids. This phenomenon becomes particularly pronounced in the RGCs during extended differentiation periods, specifically beyond 120 days (Achberger, Haderspeck, et al., 2019).

In the present study, we established a retinal organoid model for DR, with a particular focus on DRN. Clinical studies based on optical coherence tomography in diabetic patients have shown that these patients often display a progressive loss of the ganglion cell and nerve fibre layers [7]. Additionally, these patients can experience vision changes, even in the absence or presence of minimal DR [79]. Also, studies in human retinas further suggest that changes in neuronal cells, such as cell death and axon degeneration, occur before vascular abnormalities arise [7, 9]. These findings are supported by both in vitro and in vivo studies [55]. Given these findings, we believe that retinal organoids represent a valuable model for exploring the initial stages of DR, dissecting DRN without the interference of the vascular system. Moreover, we limited our study to 100-day-old organoids to circumvent the potential loss of RGCs, which is typical of advanced differentiation stages.

Our in vitro experimental design replicates the molecular features observed in DR patients by adjusting the glucose concentrations in retinal organoids medium to concentrations between 50 and 75 mM. While the glucose concentrations used in our study do not directly correspond to the serum levels found in either healthy or diabetic patients, it is important to note that our standard culture medium has a basal glucose concentration of 19 mM (control conditions). This reflects the high metabolic rate of neuronal cells in comparison to other cell types. Notably, the neuroretina is the neuronal tissue with the highest energy demand [80]. Photoreceptors, which predominantly absorb glucose in the retina, obtain their glucose supply from the choroidal blood [81]. This glucose is channelled through the basolateral and apical membranes of the RPE before reaching the photoreceptors [81, 82]. Interestingly, under the conditions we tested, photoreceptor progenitors did not show significant loss due to the high glucose concentrations. This might suggest that either a more extended treatment is necessary to observe alterations, or their metabolic requirement on glucose enables them to cope with these conditions. The loss of photoreceptors in DR might be an indirect result of other cell loss or metabolic dysregulation of the RPE-supporting cells.

Supplementing media with glucose is a classical approach used across various in vitro models. For example, dorsal root ganglion neurons required 25 mM D-glucose in a medium for optimal survival [83]. In other studies, concentrations as high as 55 mM were used to evaluate the effects of high glucose [22, 24, 84, 85]. Several other models also used glucose concentrations ranging between 30 to 55 mM, or even higher, such as the 3D in vitro model of the human cornea [86], Zebrafish embryos [87], rat retinal explants [88, 89], primary cultures of rat hippocampal neurons [90], primary cultures of rat retinal neural cells [91], and rat retinal endothelial cells [92]. Also, the duration of these treatments varies widely across studies, lasting from 2 h to 42 days (in the case of 3D in vitro model of the human cornea). This reflects different study aims, from short-term or long-term high-glucose exposure, to acute versus chronic conditions.

In diabetic patients, plasma glucose concentrations can soar to levels between 200 and 300 mg/dL (11.1 to 16.6 mM). In contrast, healthy individuals typically have fasting blood glucose levels of 99 mg/dL or lower (< 5.5 mM) (according to the Centers for Disease Control and Prevention, CDC). These changes in concentration are similar in proportion to the ones we used in our study, as the high-glucose concentrations we tested were 2.5 to 4 times higher than in the control.

Through glucose media supplementation, we have demonstrated that retinal organoids exposed to high-glucose conditions can recapitulate several characteristics observed in the early stages of DR, particularly in DRN. In these high-glucose conditions, we noted not only cell degeneration and an increase in pyknotic nuclei but also a decrease in the number of amacrine and RGCs.

Several studies have pinpointed that RGCs are the main population affected in the early stages of DR [13, 14]. The death of RGC, axonal degeneration, and ultimately optic nerve degeneration, is central in the retinal neuropathy observed in early DR. While several factors are believed to contribute to RGC malfunction and subsequent death, inflammation and oxidative stress are considered paramount [93]. In our model, we observed clear indications of glial reactivity and inflammation. Under retinal stress, both glial fibrillary acid protein (GFAP) and vimentin (ubiquitously expressed in retinal glial cells) are well-known sensitive markers for retinal gliosis [94, 95]. Since organoids differentiated at day 100 do not express GFAP [42], mainly due to the absence of mature astrocytes, we detected alterations in the morphology and immunoreactivity of vimentin, known to be expressed in Müller glia progenitors [57] in response to high-glucose treatments. Noticeably, 6 days of high-glucose treatment were also sufficient to induce an increase of MCP-1, *IL-1β*, and *VEGF* by glial progenitor cells. According to previous studies, in the early phases of DR, *VEGF* expression and release are increased, which is believed to be the retina’s protective response to prevent cell damage [96, 97]. At this stage, VEGF is a prosurvival agent rather than a proangiogenic factor. However, its role evolves as the disease progresses and VEGF levels remain high. VEGF production by Müller cells is essential for the inflammation and vascular leakage observed in DR, as evidenced in a Müller cell VEGF knockout mouse model [98].

Increased production of ROS is a well-studied consequence of high glucose levels, and it is known to be promoted by several metabolic pathways (polyol, hexosamine, AGE, and PKC pathways) [71, 72]. In our experimental setup, we also observed increased ROS levels together with an increase in the protein levels of catalase, SOD1, and SOD2, indicating that the first-line antioxidant defence is being activated. However, these increased protein levels cannot be translated into effective ROS prevention or neutralization at higher glucose levels. Indeed, it is speculated that post-translational modifications such as O-glycosyl-N-acetylation (O-GlcNAc) and glycation, which are potentiated in hyperglycaemia and diabetes, can affect antioxidant enzymes activity which was found to be decreased in DR animal models and in patients [71, 99]. Interestingly, in a Müller cell line, ROS production in response to high glucose was observed to be dependent on mTOR activity [100]. The mTOR pathway oversees various biological processes, including protein synthesis, cell proliferation, autophagy, metabolism, and cell survival. In our experiments, exposure to high glucose resulted in increased mTOR activity, correlating with a rise in pS6 levels, a key effector in the mTOR pathway. Studies in vitro and in vivo showed that mTOR signalling is amplified in streptozotocin (STZ)-injected hyperglycaemic rats and in cultured Müller cells under high-glucose conditions [100, 101]. While our findings suggest a significant role for mTOR in DR, comprehensive studies are required to fully understand its importance within the disease’s context. Future research should assess whether the molecular and cellular changes observed after 6 days of high-glucose exposure can be reversed. It would also be insightful to study the effects of prolonged high-glucose treatments, either at the same or reduced concentrations, on the neuroretina.

## Conclusions

We believe that creating a DRN model using human cells can provide a valuable platform for drug screening, in addition to rodent models. This approach could be more relevant for translational research. Additionally, both virus-based and non-virus-based gene therapy methods can be investigated using these 3D models.

Our 3D retinal model for DR opens new possibilities to study the mechanisms behind this disease. Organoids are a valuable tool to mimic disease, showing several features of DR that are independent of vascularization problems. Long periods of high glucose, more mature organoids that include other retinal cell types, and testing other hiPSCs cell lines are experimental strategies that can be further explored. Also, establishing more complex systems with vascularization and microfluidics to explore vascular mechanisms involved in disease progression presents an interesting approach.

We are confident in the potential of this model because (i) it uses human cells, yielding reliable results and promoting translational research; (ii) it offers accessibility, as a significant quantity of organoids can be produced compared to the limited number of eyes available from murine models; and (iii) it can reduce the reliance on animal testing.

## Supplementary Information

Below is the link to the electronic supplementary material.

Supplementary file1 (PDF 167 KB)

## Data Availability

The data that support the findings of this study are available from the corresponding author upon reasonable request.
