# Supplementary material for: Modelling neurodegeneration and inflammation in early diabetic retinopathy using 3D human retinal organoids
Source: In Vitro Model. 2024 Mar 25;3(1):33–48. doi: 10.1007/s44164-024-00068-1 (PMC11756505; doi:10.1007/s44164-024-00068-1)
Supplement: Supplementary file 1 — Supplementary file1 (PDF 167 KB) [file 44164_2024_68_MOESM1_ESM.pdf]

Supplementary Figure 1

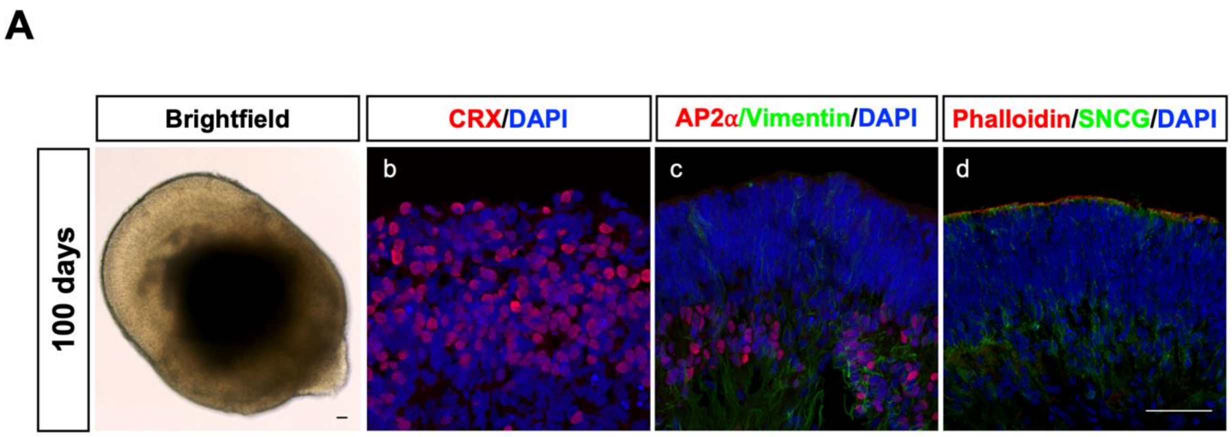

Supplementary Fig. 1 Characterization of retinal organoids derived from hiPSCs at 100 days of differentiation. **A)** Brightfield image (a) and immunofluorescence staining's (b-d) of photoreceptor progenitors (CRX), amacrine cells (AP2α), Müller glia progenitors (Vimentin), retinal ganglion cells (SNCG) and the outer limiting membrane (Phalloidin) counterstained with DAPI. Scale bar: 50 μm
